# Development and characterization of an all-in-one gamma probe with auto-peak detection for sentinel lymph node biopsy based on NEMA NU3-2004 standard

**DOI:** 10.1007/s12149-021-01581-z

**Published:** 2021-01-19

**Authors:** Aram Radnia, Hamed Abdollahzadeh, Behnoosh Teimourian, Mohammad Hossein Farahani, Mohammad Esmaeil Akbari, Habib Zaidi, Mohammad Reza Ay

**Affiliations:** 1 Research Center for Molecular and Cellular Imaging, Tehran University of Medical Sciences, Tehran, Iran; 2 Department of Medical Physics and Biomedical Engineering, Tehran University of Medical Sciences, Tehran, Iran; 3 Cancer Research Center, Shahid Beheshti University of Medical Sciences, Tehran, Iran; 4 Division of Nuclear Medicine and Molecular Imaging, Department of Medical Imaging, Geneva University Hospital, CH-1211 Geneva 4, Switzerland; 5 Geneva University Neurocenter, Geneva University, CH-1205 Geneva, Switzerland; 6 Department of Nuclear Medicine and Molecular Imaging, University of Groningen, University Medical Center Groningen, 9700 RB Groningen, Netherlands; 7 Department of Nuclear Medicine, University of Southern Denmark, 500 Odense, Denmark

**Keywords:** Intra-operative gamma probe, All-in-one, Sentinel lymph node, Performance evaluation, NEMA NU3

## Abstract

**Background:**

A gamma probe is a handheld device used for intraoperative interventions following interstitial injection of a radiotracer to locate regional lymph nodes through the external detection of radiation. This work reports on the design and performance evaluation of a novel fully integrated gamma probe (GammaPen), recently developed by our group.

**Materials and methods:**

GammaPen is an all-in-one pocket gamma probe with low weight and adequate dimensions, consisting of a detector, a control unit and output all together. The detector module consists of a cylindrical Thallium-activated Cesium Iodide [CsI (Tl)] crystal optically coupled to a Silicon photomultiplier (SiPM), shielded using Tungsten housing on side and back faces. The electronics of the probe consists of two small boards to handle signal processing and analog peak detection tasks. A number of parameters, including probe sensitivity in air/water, spatial resolution in air/water, angular resolution in air/water, and side and back shielding effectiveness, were measured to evaluate the performance of the probe based on NEMA NU3-2004 standards.

**Results:**

The sensitivity of the probe in air at distances of 10, 30, and 50 mm is 18784, 3500, and 1575 cps/MBq. The sensitivity in scattering medium was also measured at distances of 10, 30, and 50 mm as 17,680, 3050, and 1104 cps/MBq. The spatial and angular resolutions in scattering medium were 47 mm and 87 degree at 30 mm distance from the probe, while they were 40 mm and 77 degree in air. The detector shielding effectiveness and leakage sensitivity are 99.91% and 0.09%, respectively.

**Conclusion:**

The performance characterization showed that GammaPen can be used effectively for sentinel lymph node localization. The probe was successfully used in several surgical interventions by an experienced surgeon confirming its suitability in a clinical setting.

## Introduction

Many solid tumors have the potential to metastasize to regional lymph nodes. As a result, accurate detection of these metastases is critical in staging, prognosis and development of treatment plans [1–3]. Various strategies were devised for sentinel lymph node (SLN) detection with different levels of accuracy and degrees of success [4, 5]. A gamma probe is one of the most effective devices currently used for the detection and localization of SLN [6, 7] in breast and masculine types of cancers where the detection of sentinel nodes is mandatory [8–10]. Different radionuclides emitting at various energies (140–511 keV) are used for this purposes for tracing procedures [11].

The basic physical performance of a gamma probe depends on the chosen detector material, detector size and collimation [12–15]. These specifications affect the performance of a gamma probe in terms of technical parameters, such as sensitivity, side and back shielding, angular resolution and spatial resolution, which in turn impact the successful identification of sentinel lymph nodes [16]. A high sensitivity is required for deep-seated nodes and low tracer uptake detection; proper spatial resolution and angular resolution are important for distinguishing nodes close to injection site or close to each other; good shielding is mandatory for omitting unwanted radiation from other directions. The sensitivity, angular resolution, and spatial resolution are commonly evaluated in scatter medium. Hence, accurate photopeak detection is important for removing unwanted scattered photons. Various methods were reported for performance evaluation of gamma probes [13, 17], with the most popular being the NEMA NU3-2004 standard [11].

Current gamma probes include a probe connected with a long wire to a console where there are many other devices and wires in the surgery room. These operating conditions are not suitable for daily routine activities of surgeons. The idea behind designing GammaPen was the removal of the wires and console and their integration inside the probe. In this work, we report on an innovative compact design and construction of GammaPen which provides highly accurate peak detection and measurement beside the small dimensions of the electronic boards. The specifications of GammaPen based on NEMA NU3-2004 standard are also reported.

## Materials and methods

### GammaPen design

**Fig. 1:**
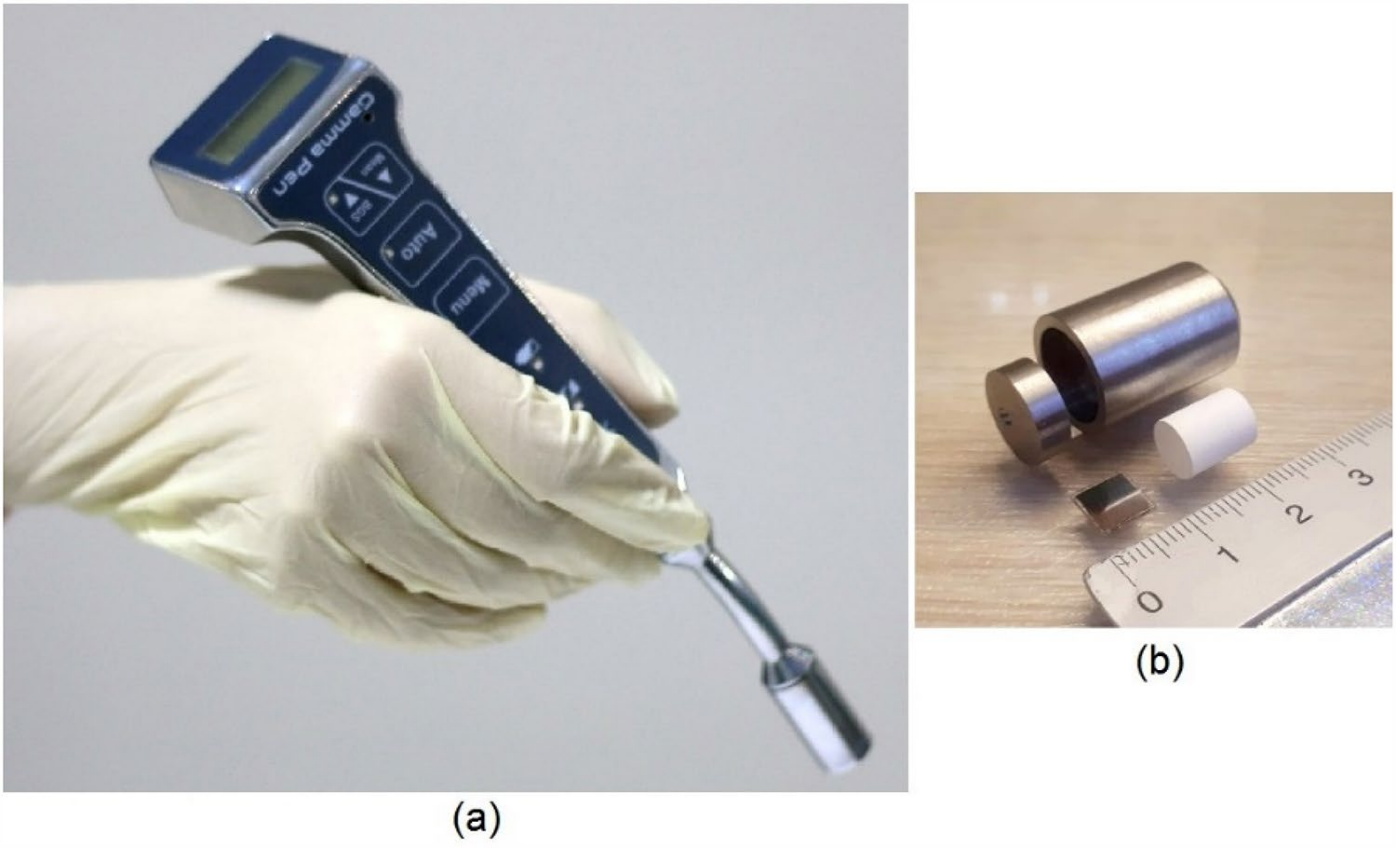
**a** The GammaPen system and **b** the detector module consisting of Tungsten shielding, crystal and photodiode

We developed GammaPen, a rechargeable wireless gamma probe dedicated for sentinel lymph node biopsy (SLNB) (Fig. 1a). This all-in-one system consists of a head with its associated detection parts encapsulated in an ergonomic housing, two compact boards for signal processing and peak detection, and input/output parts for control, display, and sound.

#### Head and body design

The GammaPen head comprises CsI (Tl) scintillation crystal (Epic Crystal Co., China) with 7.5 mm diameter and 10 mm length coupled to a MicroFC-60035-SMT SiPM (ON Semiconductor Co., USA), with 6 × 6 mm active area [18]. We designed the crystal with 7.5 mm diameter for efficient use of the active area of the SiPM. The GammaPen is primarily optimized for use with 140 keV gamma rays (the dominant gamma energy of Tc-99m). Yet, it can be also used for gamma-ray energies ranging between ∼60 keV (Am-241) and 364 keV (I-131).

The SiPM has proper photon detection efficiency (PDE) between 350 and 550 nm, having good overlap with CsI (Tl) emission spectra which has a high intensity between 480 and 630 nm [18, 19]. The detector is equipped with a Tungsten pinhole collimator with 7.5 mm internal diameter, 2.8 mm lateral thickness, and 225 mm length (Fig. 1b). The detector modules are shielded using 2.8-mm-thick tungsten for side and back faces and placed in a housing. The head tip diameter is about 14.5 mm.

**Fig. 2:**
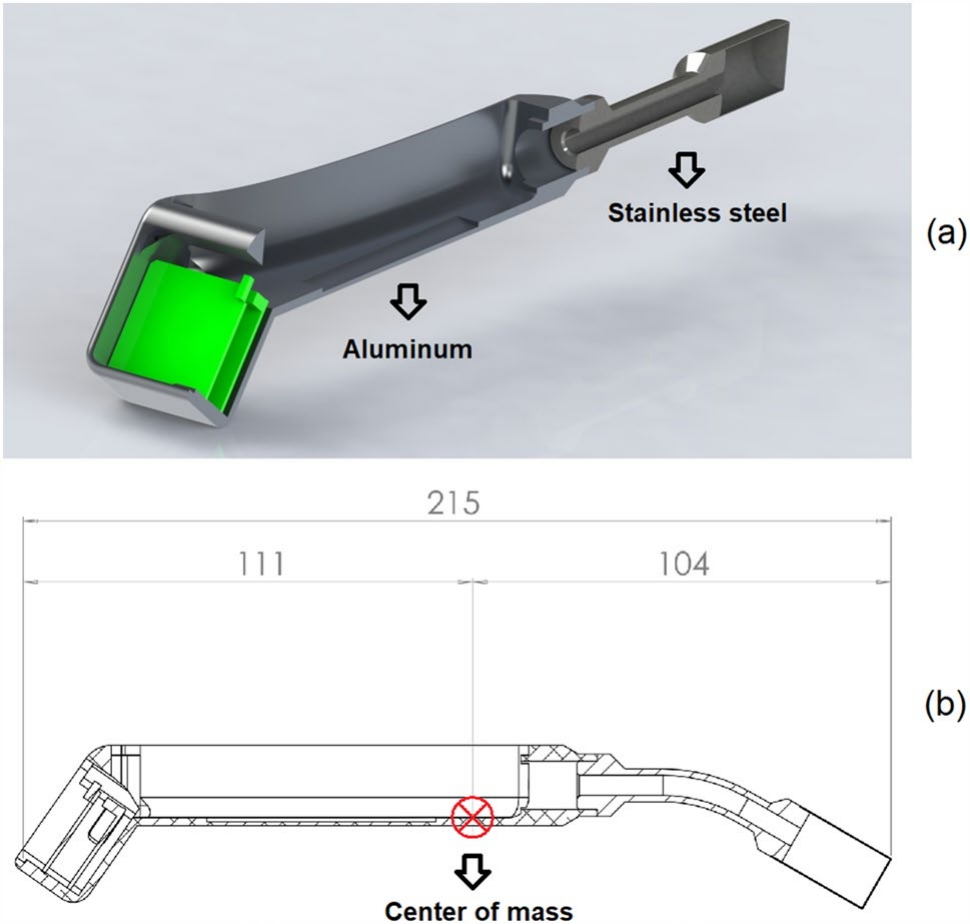
**a** The GammaPen body design and **b** the position of the center of mass

The body of GammaPen consists of two machined parts. The first one is the head housing made of stainless steel whereas the second one is the housing of electronic components made of aluminum (Fig. 2a). The overall weight of these two parts is about 100 g with the center of mass showed in Fig. 2b. The position of the center of mass is near the center of the body which makes the device more stable in hand. The device weight consisting of body, Tungsten, battery and other electronic components is about 170 g, which is a suitable weight for handling. As a result, based on the weight, the center of mass and shape of the body, the device is ergonomic and easy to handle.

#### Signal processing and peak detection

**Fig. 3:**
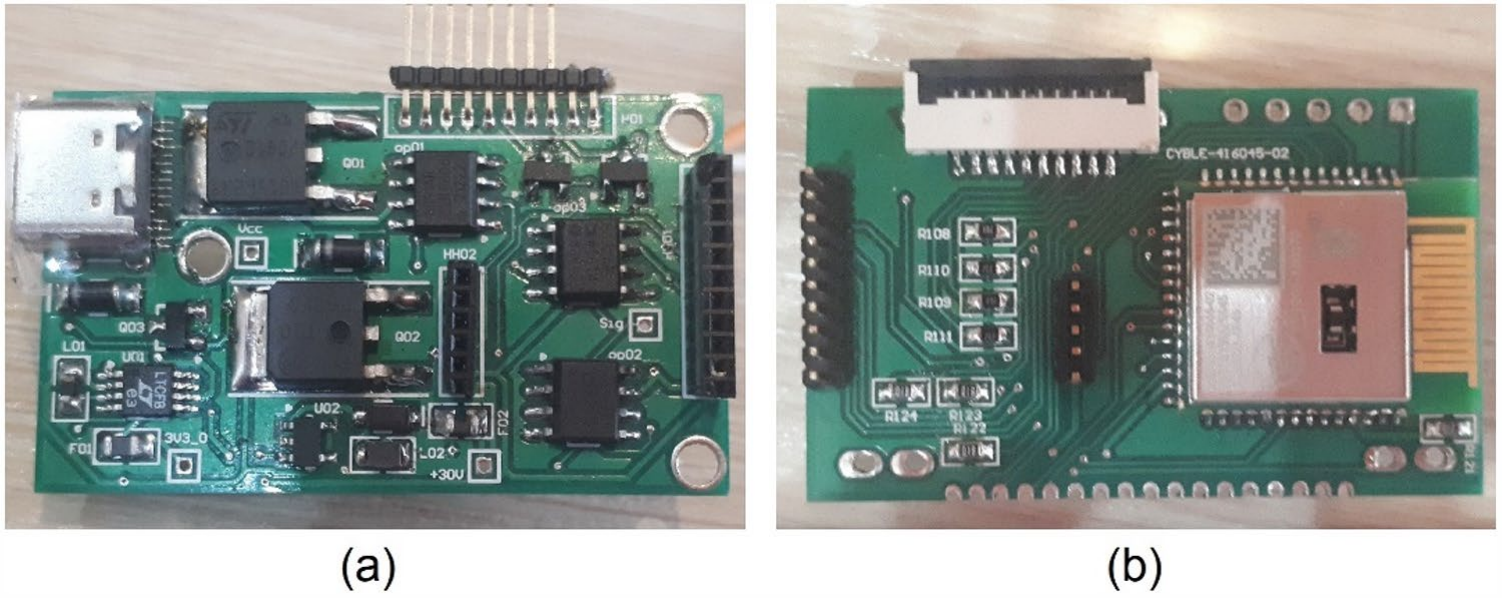
The electronics consist of two boards: **a** the first one for analog signal processing and voltage regulation. **b** The second one for micro-controller and display

**Fig. 4:**
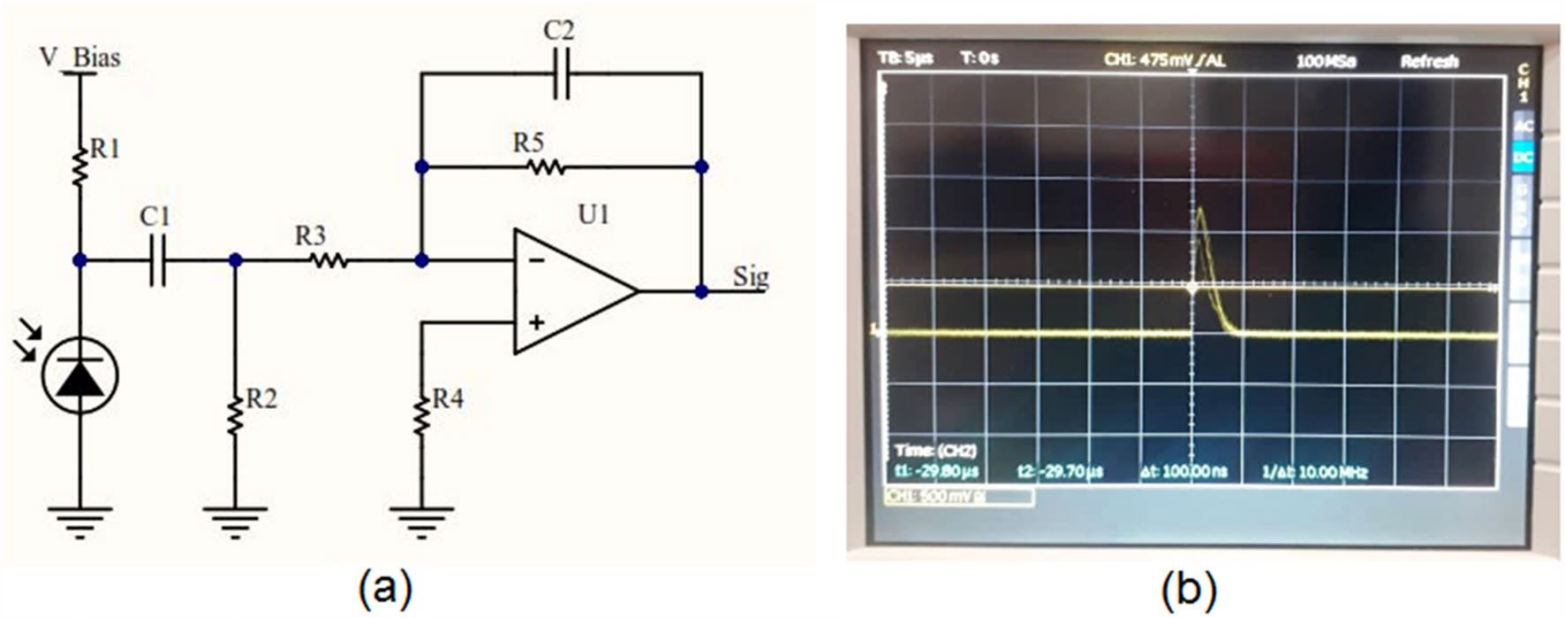
Simplified circuit schematic of signal pre-amplification along with the signal measured with HAMEG oscilloscope (A Rohde and Schwarz Company)

Dedicated electronics consists of two boards developed for signal processing and data acquisition placed inside the stainless-steel probe’s body (Fig. 3). The electronics provides a 30 V bias voltage for the SiPM. Signal pre-amplification is performed in the electronic board with gain of 10 (Fig. 4a). A typical pre-amplified signal is shown in Fig. 4b. In the next step, the signal’s amplitude is compared with two voltage levels controlled by micro-controller variable current digital to analog converter (DAC) output. The low power micro-controller module used in this design is CYBLE-416045 (Cypress Semiconductor Co., USA) consisting of PSOC-63-BLE of ARM family. The PSOC-63 micro-controllers (Cypress Semiconductor Co., USA) operate at high speed and support Bluetooth low energy (BLE). Another noteworthy option of this module is its current DAC output. We use this current DAC output for setting two voltage thresholds for peak detection. R6 and R7 are fixed so that the energy resolution is also fixed (Fig. 4), but with changing the current DAC output, we can collect counts at different energies. In calibration mode with a fixed radiation source, the micro-controller changes IDAC value in one-second steps for finding the peak of energy, then the IDAC value of the peak is fixed for future measurements. In previous designs of gamma probes, it was common to use high speed analog to digital converters (ADCs) for live integration and peak detection, while in this new innovative design, there is no need for time-consuming procedures of live integration.

**Fig. 5:**
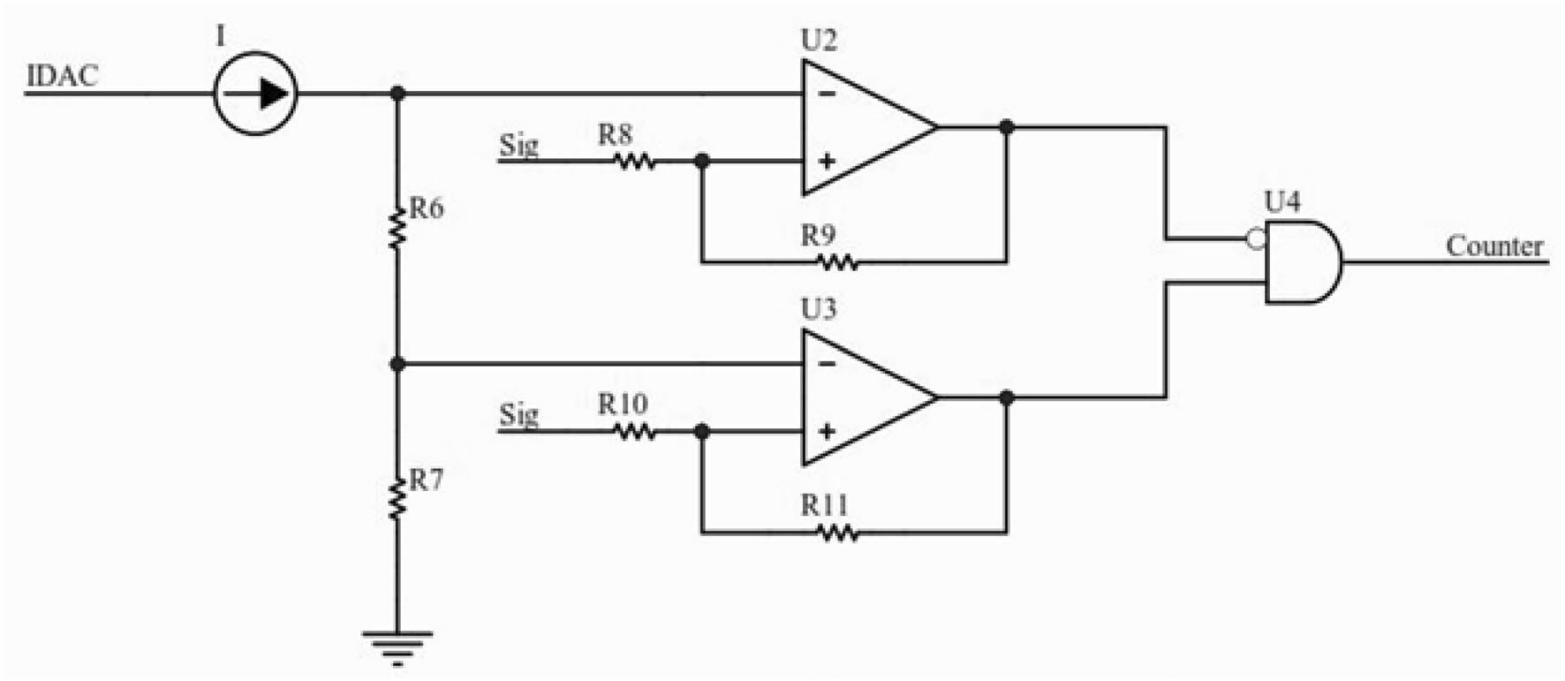
Simplified circuit schematic of analog peak detection

U2 and U3 Op-Amps are high frequency with high input impedance, acting as triggers to help selecting signal amplitudes between the two voltage levels. At the end, after AND gate, we count pulses to measure signals between these two voltage levels (Fig. 5). The proportion of R6/R7 has direct relation with energy resolution. In the current design, R6 and R7 are calculated for 12% energy window.

#### Control and output unit (key pad, display, sound and Bluetooth)

The control unit is a built-in user-friendly system equipped with dot matrix LCD, audio sound generator featuring user-selectable count rate scale, isotope selection among Am-241, Tc-99m, and I-131, audio signal with adjustable volume, add-up option for displaying the average counts for 10 s acquisition, and finally a background suppression mode. In background suppression mode, the background radiation rate is measured for 10 s and then subtracted from the total collected counts. GammaPen also supports Bluetooth low-energy (BLE) for connecting to a mobile or tablet providing big display for users who prefer large count rate display and high range volume as an option in situations, such as SLNB workshops.

### Performance evaluation

**Fig. 6:**
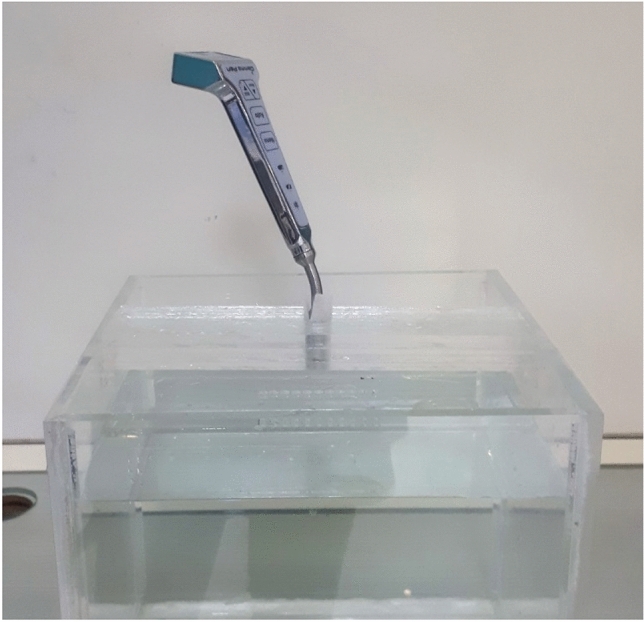
Experimental measurements setup for NEMA NU3 evaluation in a scattering medium using a water bath with dimensions of 25 cm long × 25 cm wide × 20 cm deep

The performance characteristics of GammaPen were measured using NEMA NU3-2004 standard [11]. The radiation source used for this purpose was Tc-99m solution with 0.362 MBq activity in the form of a point-like source in a 1 mm diameter capillary tube using rubber like silicon paste to confine the activity volume. For tests in air, the source-to-probe centerline was at least 50 mm far from any scattering material while, for tests in scattering medium, measurements were performed in a 25 cm long × 25 cm wide × 20 cm deep water-filled container (Fig. 6) where the source was placed at a certain depth of water and the probe positioned such that its tip touches the water surface.

#### Sensitivity in air and scattering medium

For sensitivity measurements, the source was aligned with the central axis of the field-of-view (FOV) in front of the head tip. Then, we measured the sensitivity for source distances ranging from 10 to 50 mm with 20 mm steps. More than 10,000 counts were recorded for each position. The sensitivity was then calculated as counts per second per unit of radioactivity at a specific distance.

#### Spatial resolution in air and scattering medium

**Fig. 7:**
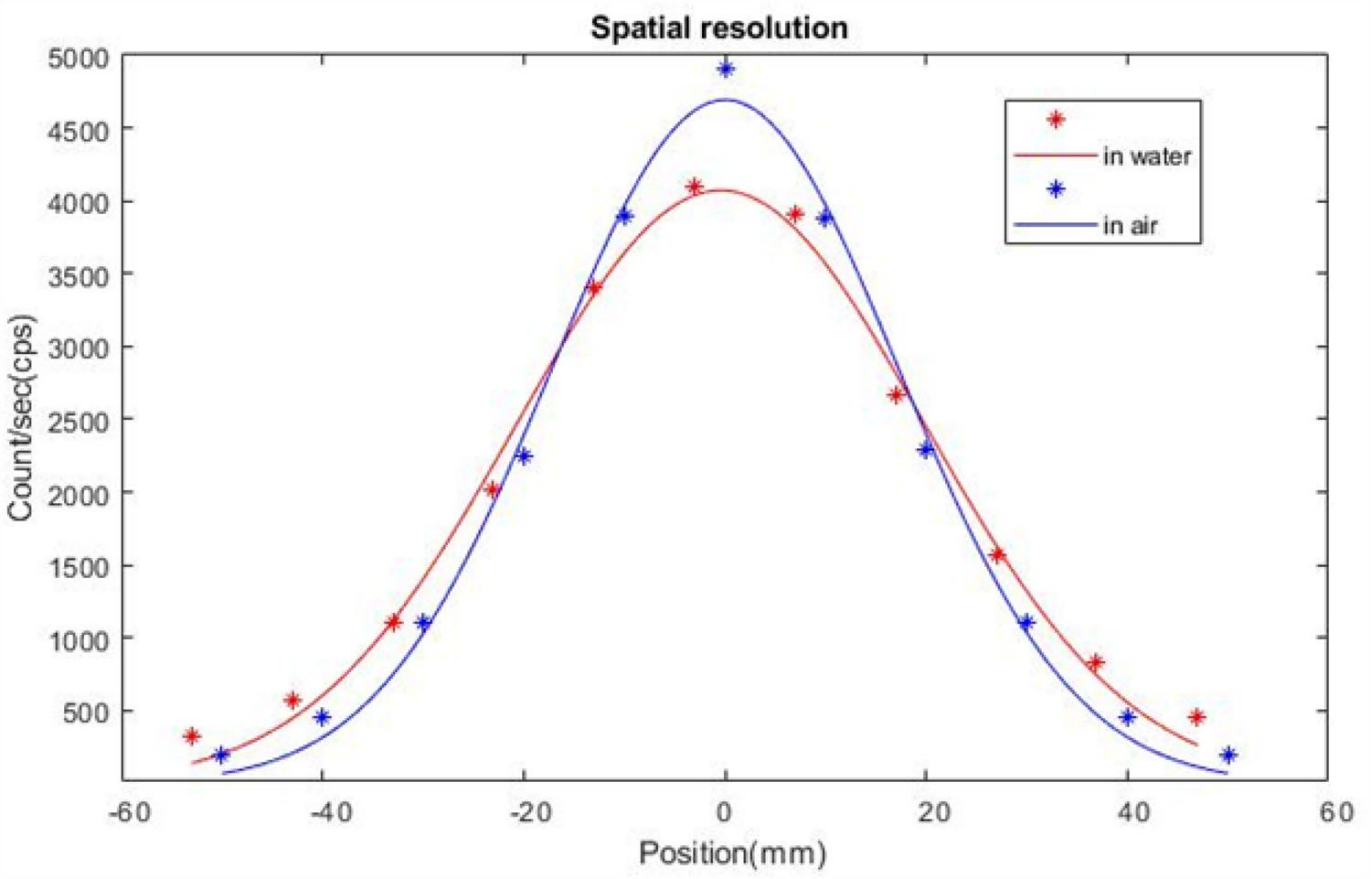
Spatial resolution profile of GammaPen for a point source at 30 mm distance in water and in air

To measure the spatial resolution, the radioactive source was positioned along the central axis of the probe at 30 mm distance. While maintaining this distance constant, the distance between the source and the probe axis was changed from − 50 mm to + 50 mm using 10 mm steps. The source activity was low enough to produce a count rate within the linear count rate response region of the system. Using the measured data, the spatial resolution in scattering medium was reported in terms of full-width at half-maximum (FWHM) at 30 mm probe-to-source distance. Figure 7 shows Gaussian curves fit of measured data for spatial resolution in water and in air.

#### Angular resolution in air and scattering medium

**Fig. 8:**
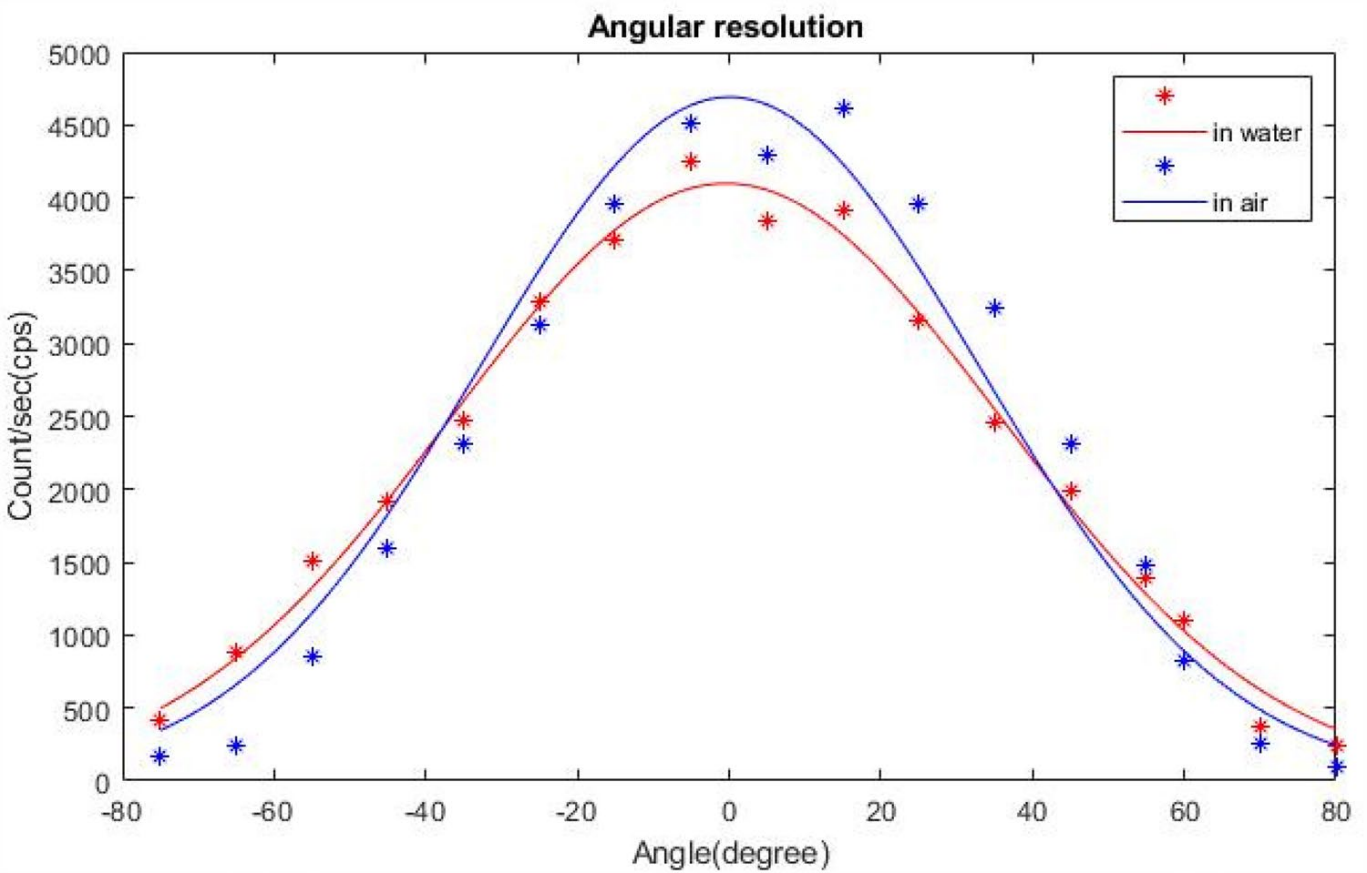
Angular resolution profile of GammaPen for a point source at 30 mm distance in water and in air

Using the same above-described setup for spatial resolution measurement, we kept the radioactive source at a fixed distance of 30 mm. The probe was clamped and rotated about the center of the probe window in 10° steps. The probe was oriented at different angles from the source in the range − 80° to + 80°. Using the measured data at different angles, the angular resolution was reported in terms of FWHM. Figure 8 shows Gaussian curves fit of measured data for angular resolution in water and in air.

#### Shielding effectiveness and leakage sensitivity

The side and back shielding behavior was measured in air using a Tc-99m point source with 20 MBq activity. First, the point source was placed in front of the probe touching the detector surface to measure the axis count rate (CPS_Axis_). Subsequently, it was slowly moved around the entire outer surface of the body touching the probe and the maximum observed count rate considered as the leakage count rate (CPS_Leak_). The side and back shielding was then calculated as the count rate for unit of radioactivity using equations below:

1 $$\mathrm{Shielding effectiveness }\left(\mathrm{\%}\right)=\frac{\left(\mathrm{CPS}/{\mathrm{MBq}}_{\mathrm{axis}}-\mathrm{CPS}/{\mathrm{MBq}}_{\mathrm{leak}}\right)}{\mathrm{CPS}/{\mathrm{MBq}}_{\mathrm{axis}}},$$

2 $$\mathrm{Leak sensitivity }(\mathrm{\%}) =\frac{\mathrm{CPS}/{\mathrm{MBq}}_{\mathrm{leak}}}{\mathrm{CPS}/{\mathrm{MBq}}_{\mathrm{axis}}}$$

## Results and discussion

The GammaPen performance based on NEMA NU3-2004 standard is summarized in Table 1. The sensitivity of GammaPen in air at 10, 30, and 50 mm distances is 18784, 3500, and 1575 cps/MBq, respectively. As expected for the single-hole collimation, the sensitivity decreases inversely proportional to the squared distance. The sensitivity in scattering medium was also measured at 10, 30, and 50 mm distances as 17,680, 3050, and 1104 cps/MBq. As expected, the sensitivity in scatter medium is less than the sensitivity in air.

**Table 1:** GammaPen performance parameters measured experimentally based on NEMA NU3 protocol

Measured parameter	Value	Uncertainty	Distance	Medium
Sensitivity (cps/MBq)	18,780 17,680 3500 3050 1570 1100	± 1% ± 1% ± 1% ± 1% ± 1% ± 1%	10 mm 10 mm 30 mm 30 mm 50 mm 50 mm	Air Scatter Air Scatter Air Scatter
Spatial resolution FWHM (mm)	40 47	± 1% ± 1%	30 mm 30 mm	Air Scatter
Angular resolution FWHM (°)	77° 87°	± 1% ± 1%	30 mm 30 mm	Air Scatter
Shielding effectiveness (%)	99.91	± 0.01		Air
Leak sensitivity (%)	0.09	± 0.01		Air

The sensitivity is one of the most important parameters for lymph node detection to depict low-uptake or deep-seated nodes. Since sentinel nodes are mostly located at about 30 mm depth of the body surface [20–24], the gamma probe should provide proper sensitivity at 30 mm distance, to give useful information about the SLN in a practical acquisition time. The sensitivity of GammaPen is about 3050 and 3500 cps/MBq at 30 mm distance from the collimator in scatter medium and air, respectively. Our results also confirm that the sensitivity is inversely proportional to the square of the distance from the probe [25, 26]. The energy window of the system is set to 12%. As a result, the sensitivity in the scattering medium decreases with respect to sensitivity in air by 5.6, 12.8, and 29.9% at 10, 30, and 50 mm distance, respectively. Hence, most of the scattered photons are rejected revealing insensitivity of GammaPen to scattered radiation.

Figures 7 and 8 shows the spatial resolution and the angular resolution profiles in scatter medium and air for a point source at 30 mm depth. The high spatial resolution of the gamma probe enables accurate identification of lymph nodes sited near each other and also nodes close to the injection site [10]. The spatial resolution and sensitivity are highly dependent on the characteristics of the collimator and the crystal [27]. Improving one of these parameters results in worsening the other, hence, the sensitivity and spatial resolution of gamma probes should be optimized depending on the envisaged clinical applications [10]. The results shown in Table 1 show that the spatial resolution and angular resolution in scatter medium are 47 mm and 87 degree at 30 mm distance from the probe in scatter medium, while they are 40 mm and 77 degree in air, respectively. This difference is due to the presence of scattering medium and this difference increases with increasing the energy window span. In scatter medium, there are more Compton photons in comparison with in air. Therefore, when the energy window is wider, more Compton photons will be counted from different angles, and as consequence the spatial resolution and angular resolution will degrade in comparison with air.

The detector shielding effectiveness and leakage sensitivity are 99.91% and 0.09%, respectively, for GammaPen. It is important for gamma probes to have low shielding leakage, because a weak shielding may cause detecting unwanted photons from out of the field-of-view (FOV), which might mislead the surgeon [10, 28, 29].

A suitable gamma probe in clinical environment should have a high sensitivity and good shielding as well as good spatial and angular resolution [30]. However, there is no specific probe with optimal performance for all mentioned parameters to be used for all surgical applications. As a consequence, there are different configurations of probe size and collimator to be used in different applications. For example, when the predominant use of a gamma probe is for SLN biopsy in patients with breast cancer or melanoma, the sensitivity is the most important parameter [31, 32]. In fact, it is crucial for the gamma probe to be able to detect lymph nodes with low uptake. Conversely, although a high spatial resolution is desirable, it is relatively less important than the sensitivity in SLN procedures. The NEMA NU3 standard provides a reference for comparing the performance parameters of various gamma probes. Table 2 shows the parameters of different commercially available systems based on NEMA NU3 standard. As can be observed, Europrobe (Eurorad company) and Neoprobe (Neoprobe company) without an additional external collimator provide a higher sensitivity, while probes using an additional external collimator (C-Track, Navigator) have poorer sensitivity [12, 17]. GammaPen with thinner collimator has a high sensitivity and comparable spatial resolution and angular resolution with other probes that do not utilize an external collimator. It should be noted that the side and back shielding of the GammaPen is better [11, 12].

**Table 2:** NEMA NU3 results of GammaPen and some available commercial gamma probes [17]

Parameter	Value			
	GammaPen with collimator	C-track with collimator	Europrobe without collimator	Europrobe large with collimator
Diameter (mm)	14	15	16	19
Sensitivity at 30 mm in air (cps/MBq)	3500	1500 ± 200	1900 ± 300	1240 ± 190
Sensitivity at 50 mm in air (cps/MBq)	1575	680 ± 100	770 ± 120	560 ± 80
Sensitivity at 30 mm in scatter (cps/MBq)	3050	900 ± 150	1700 ± 300	920 ± 140
Sensitivity at 50 mm in scatter (cps/MBq)	1104	400 ± 60	600 ± 90	330 ± 50
Spatial resolution FWHM at 30 mm (mm)	47	28	43	22
Angular resolution FWHM at 30 mm (deg)	87	61	102	46
Shielding maximum leakage (cps/MBq)	70	0.8 ± 0.2	17.0 ± 0.7	0.06 ± 0.04
Shielding maximum leakage (%)	0.09	0.02	0.15	0.003

## Conclusion

In this work, we described the design considerations and performance evaluation of GammaPen recently developed by our group based on NEMA NU3-2004 standard. The innovative electronic design for signal processing and peak detection resulted in a light-weight compact structure with small dimension beside low power consumption and high accuracy. Eventually, this easy to use and portable all-in-one pocket gamma probe consisting of a detector, control unit and output all together, showed high performance for SLNB applications.

The designed gamma probe provided a high sensitivity (about 3500 and 3050 cps/MBq in air and scatter medium, respectively) and a spatial resolution of about 40 and 47 mm in air and scatter medium, respectively. An angular resolution of about 77 and 87 degree in air and in scatter medium, respectively. All parameters were measured at a distance of 30 mm from the collimator. The shielding effectiveness was more than 99.91%. The measured performance characteristics of GammaPen showed that it can be used with confidence for sentinel lymph node identification during radiosurgery. The probe was successfully used in several surgical interventions.
